# Supplementary material for: High‐throughput identification of RNA nuclear enrichment sequences
Source: EMBO J. 2018 Jan 15;37(6):e98452. doi: 10.15252/embj.201798452 (PMC5852646; doi:10.15252/embj.201798452)
Supplement: Supplementary file 1 — Appendix [file EMBJ-37-e98452-s001.pdf]

High-throughput identification of RNA nuclear enrichment sequences

Chinmay J Shukla, Alexandra L McCorkindale, Chiara Gerhardinger, Keegan D Korthauer, Moran N Cabili, David M Shechner, Rafael A Irizarry, Philipp G Maass, John L Rinn

Table of Contents:

Supplementary Figure Legends	2
Supplementary Figure 1	3
Supplementary Figure 2	4
Supplementary Figure 3	5

Supplementary Figure Legends:

Figure S1. Sequence Features of Differential Regions A. UCSC genome browser tracks showing the overlap of *XIST* repeats and DRs. **B.** Boxplot showing the length distribution of the differential regions (DRs) identified by the MPRNA across all tested lncRNAs. Most detected DRs were longer than the tiled 110-nt oligo nucleotide. **C.** A scatter plot showing the relationship between number of DRs in a lncRNA (X-axis) and the length of the lncRNA (Y-axis). The blue line shows the loess fit and the shaded region the confidence interval around the fit. **D.** Comparison of GC content between DRs and non DRs shows no noticeable difference.

Figure S2. Motifs enriched in lncRNA nuclear enrichment signals. A-D. Position Weight Matrices (PWMs) for motifs enriched whithin DR sequences. The motif in panel A is similar to the C-rich motif. Motifs in panels B-D are found in *XIST* and are similar to the specific *XIST* motif (see **Fig 4A,** E-Value < 0.05). **E.** k-mers are mildly predictive of DRs found using ridge regression. The color describes the weight of the k-mer assigned by the ridge regression algorithm (see *Materials and Methods)*.

Figure S3. Novel C-rich motif can influence the localization of endogenous human transcripts. Comparison of nuclear enrichment of all human transcripts with at least one occurrence of our discovered motifs to all other transcripts, in all ENCODE Tier 2 cells(An Integrated Encyclopedia of DNA Elements in the Human Genome, 2012). *P*-value: Mann Whitney Test.

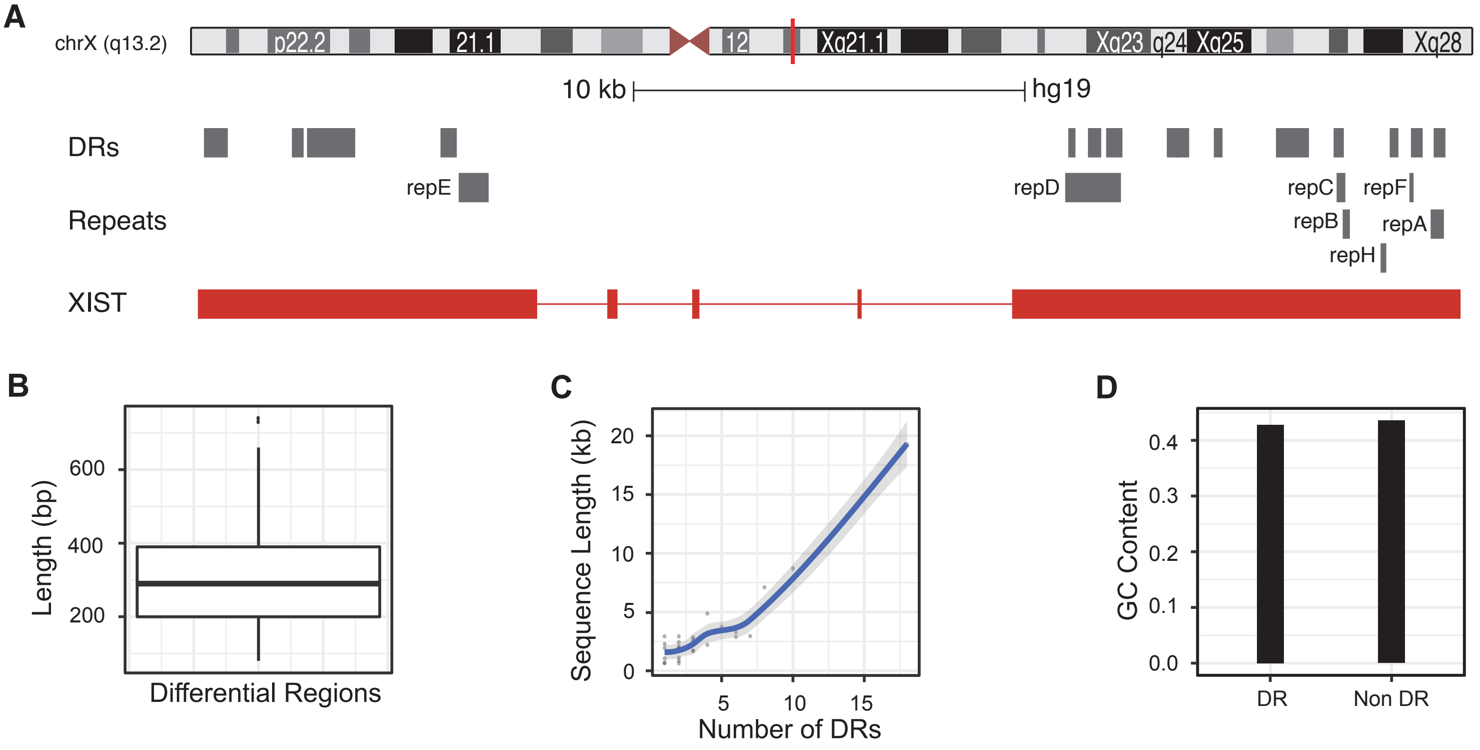
A
chrX (q13.2)
p22.2
21.1
12
Xq21.1
Xq23
q24
Xq25
Xq28
10 kb
hg19
DRs
repE
repD
repC
repF
Repeats
repB
repA
repH
XIST
B
Length (bp)
600
400
200
Differential Regions
C
Sequence Length (kb)
20
15
10
5
0
5
10
15
Number of DRs
D
GC Content
0.4
0.3
0.2
0.1
0.0
DR
Non DR

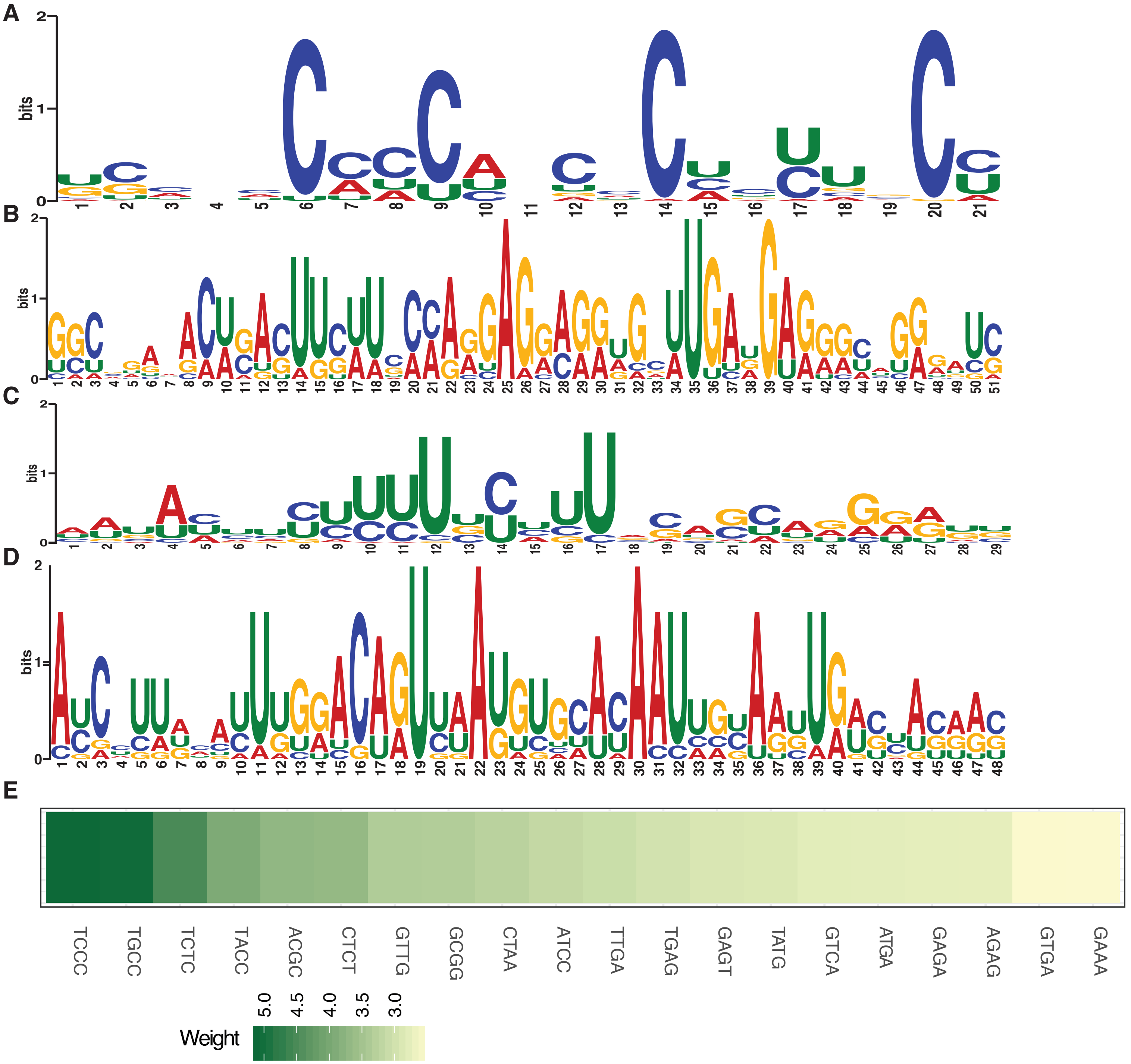
A
B
C
D
E
bits
TCCC
TGCC
TCTC
TACC
ACGC
CTCT
GTTG
GCGG
CTAA
ATCC
TTGA
TGAG
GAGT
TATG
GTCA
ATGA
GAGA
AGAG
GTGA
GAAA
Weight
5.0
4.5
4.0
3.5
3.0

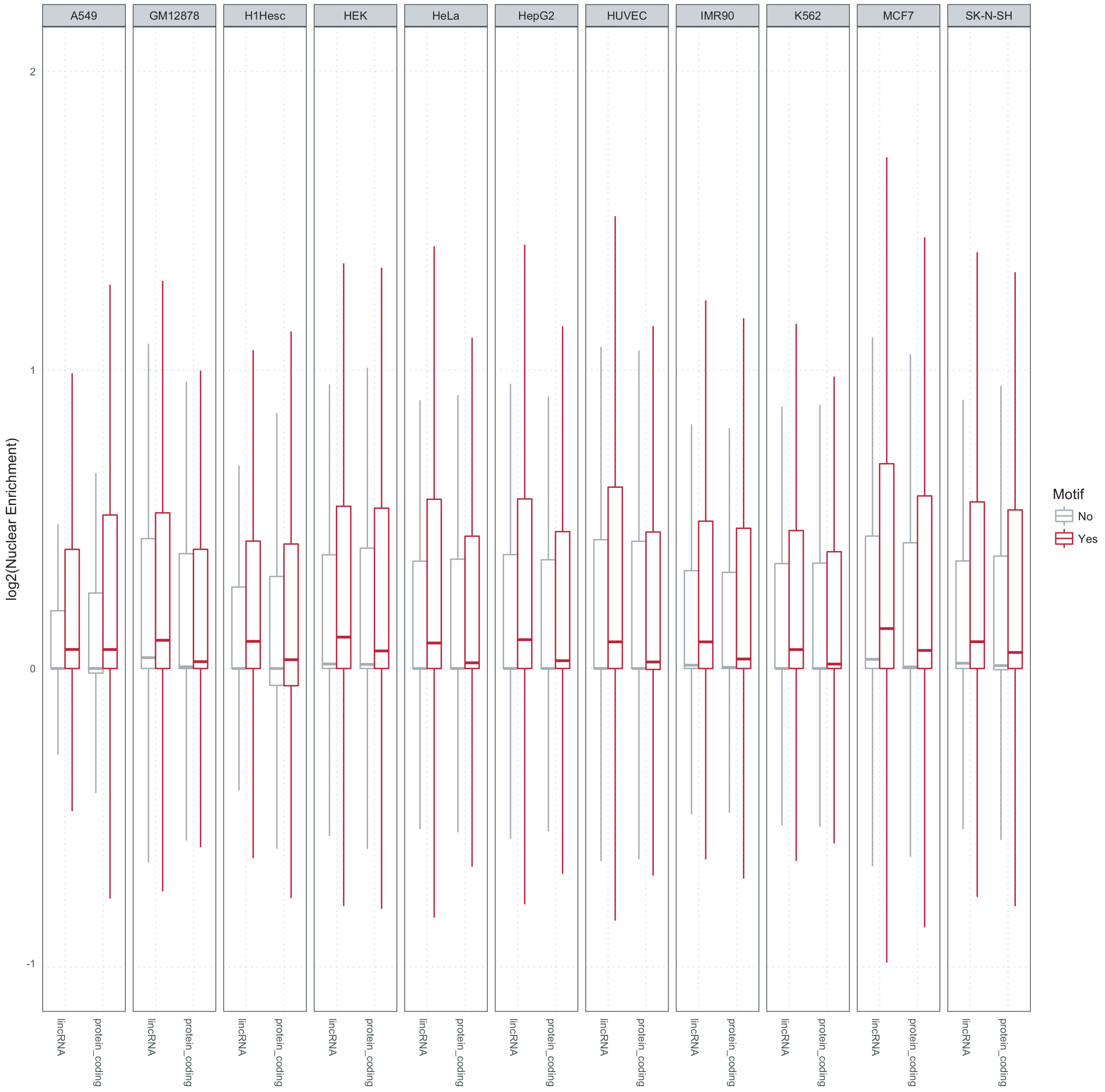

A549
GM12878
H1Hesc
HEK
HeLa
HepG2
HUVEC
IMR90
K562
MCF7
SK-N-SH
log2(Nuclear Enrichment)
2
1
0
-1
lincRNA
protein_coding
Motif
No
Yes
