# Supplementary material for: High‐throughput identification of RNA nuclear enrichment sequences
Source: EMBO J. 2018 Jan 15;37(6):e98452. doi: 10.15252/embj.201798452 (PMC5852646; doi:10.15252/embj.201798452)
Supplement: Supplementary file 2 — Expanded View Figures PDF [file EMBJ-37-e98452-s002.pdf]

Expanded View Figures

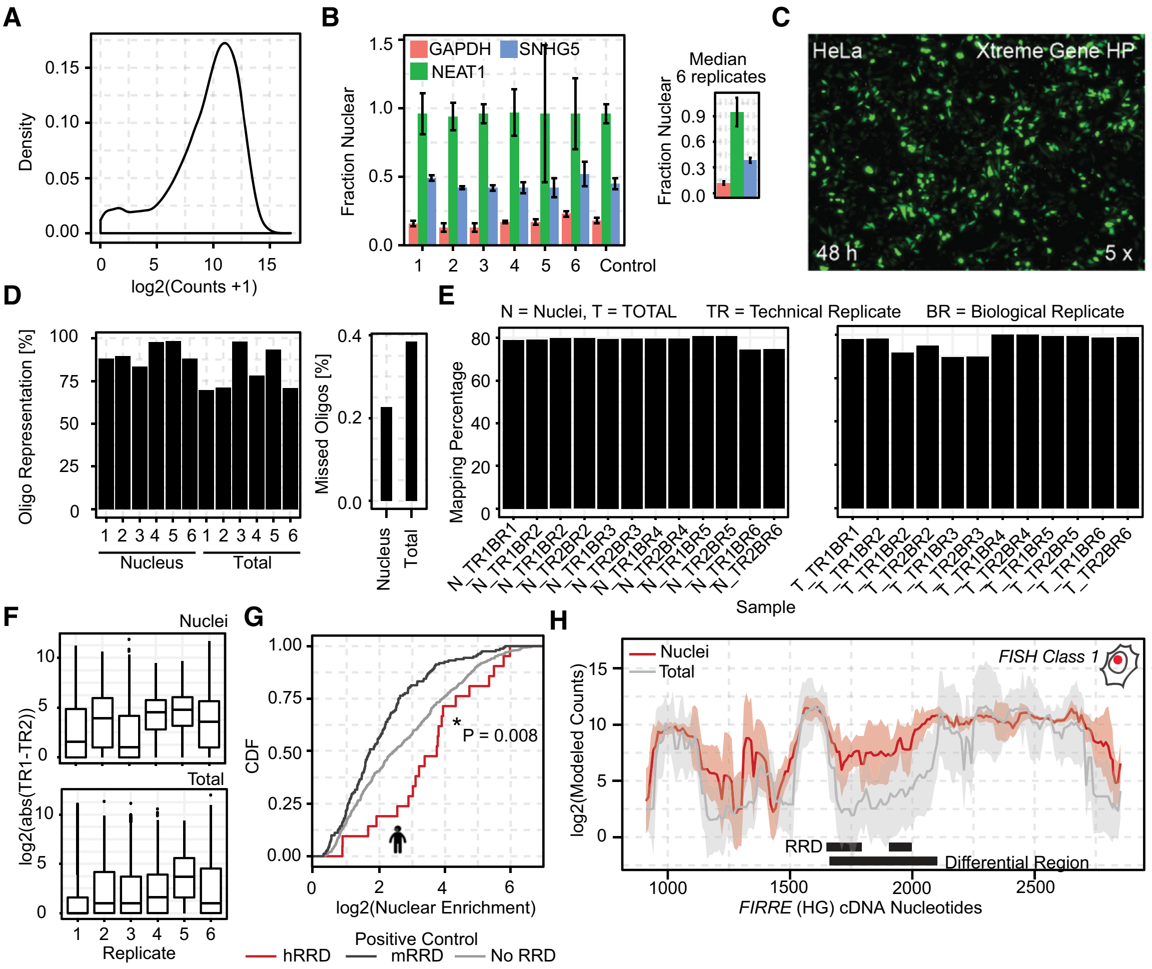

Figure EV1. Quality assessment of every MPRNA step.

A The distribution of oligos in the cloned plasmid pool. (i) the single peak showing uniform counts for several different oligos indicates very little jackpotting, and (ii) entire oligo representation (small bump at zero counts).

B Nuclear enrichment of *NEAT1*, *GAPDH*, and *SNHG5* as determined by qRT–PCR in each biological replicate (left) (error bars: ±SD). Nuclear enrichment of median ± SD across the six replicates.

C Transfection efficiency of HeLa cells co-transfected with a GFP plasmid using the protocol outlined in Materials and Methods.

D A recovery of > 70% of our initial oligo-pool was obtained in each sample. On average, only 0.2% of the oligos (i.e., ~25 oligos) was not detected in the nuclear fraction samples, and ~0.4% (i.e., ~50 oligos) in the Total (whole-cell lysate) samples.

E Bar plots showing the mapping percentage for all reads of different samples from nuclear (N) and total fractions (T), separated for technical replicates (TR) and biological replicates (BR).

F Boxplots showing inter-replicate differences between counts of the same oligo. Low technical variance was detected as indicated by low differences in counts among technical replicates. The solid horizontal line is the median while the lower and upper hinges correspond to the first and third quartiles (the 25th and 75th percentiles). The upper whisker extends from the hinge to the largest value no further than 1.5 × IQR from the hinge. The lower whisker extends from the hinge to the smallest value at most 1.5 × IQR of the hinge. Data beyond the end of the whiskers are outliers and are plotted individually.

G CDF plot of the nucleotides overlapping human RRD, mouse RRD, and other nucleotides in the human and mouse *FIRRE* loci. Similar to the *MALAT1* Region M and Region E, the MPRNA recapitulated the function of the known RNA nuclear retention element RRD of the *FIRRE* locus. Since the experiments were performed in human HeLa cells, human *FIRRE* RRD was nuclear enriched, while the mouse *FIRRE* RRD did not influence nuclear enrichment of *fsSox2* (*P*-value: Mann–Whitney test comparison between RRD nucleotides of the human FIRRE transcript versus other non RRD nucleotides in the human and mouse FIRRE transcripts).

H Differential region-calling correctly identified nuclear retention elements in *FIRRE*. Solid lines: per-nucleotide abundances in the nuclear (red) and whole-cell (gray) fractions, modeled for each position along the *FIRRE* transcript, based on the aggregate behavior of all oligos containing that nucleotide (shaded regions ±SD, medians for six biological replicates).

 © 2018 The Authors
